# Resveratrol induces H3 and H4K16 deacetylation and H2A.X phosphorylation in *Toxoplasma gondii*

**DOI:** 10.1186/s13104-020-05416-4

**Published:** 2021-01-07

**Authors:** Susana M. Contreras, Agustina Ganuza, María M. Corvi, Sergio O. Angel

**Affiliations:** 1 Laboratorio de Parasitología Molecular, Instituto Tecnológico de Chascomús (INTECH), Consejo Nacional de Investigaciones Científica Y Técnicas (CONICET), Universidad Nacional General San Martín (UNSAM), Int. Marino Km 8.3, Provincia de Buenos Aires, Chascomús, C.P. 7130 Argentina; 2 Laboratorio de Bioquímica y Biología Celular de Parásitos, INTECH, CONICET/UNSAM, Chascomús, Provincia de Buenos Aires, Argentina

**Keywords:** *Toxoplasma gondii*, Resveratrol, Treatment, Histone deacetylase, Chromatin remodeling, H2A.X, DNA damage

## Abstract

**Objective:**

Resveratrol (RSV) is a multitarget drug that has demonstrated activity against *Toxoplasma gondii* in macrophage and human foreskin fibroblast (HFF) cell line infection models. However, the mechanism of action of RSV has not yet been determined. Thus, with the aim of identifying a possible mechanism of the anti-*T. gondii* activity of this compound, we analyzed the effects of RSV on histones H3 and H4 lysine 16 acetylation (H4K16). We also analyzed RSV-induced DNA damage to intracellular tachyzoites by using the DNA damage marker phosphorylated histone H2A.X (γH2AX).

**Results:**

RSV inhibited intracellular *T. gondii* tachyzoite growth at concentrations below the toxic threshold for host cells. The IC_50_ value after 24 h of treatment was 53 μM. RSV induced a reduction in H4K16 acetylation (H4K16ac), a marker associated with transcription, DNA replication and homologous recombination repair. A similar deacetylation effect was observed on histone H3. RSV also increased *T. gondii* H2A.X phosphorylation at the SQE motif (termed γH2A.X), which is a DNA damage-associated posttranslational modification. Our findings suggest a possible link between RSV and DNA damage or repair processes that is possibly associated with DNA replication stress.

## Introduction

*Toxoplasma gondii* is an important pathogen affecting animal and human health, particularly during pregnancy and in immunocompromised patients [1]. In humans, infection starts the asexual cycle, which also occurs in other mammals and birds. The asexual phase is characterized by two stages: the rapidly replicating and highly disseminating tachyzoite and the bradyzoite. The bradyzoite replicates slowly and is located in tissue cysts for the remainder of the life of the host [2]. The current therapy against toxoplasmosis is effective but has adverse effects [3]. In recent years, with the aim of finding new therapies, various therapeutic targets have been explored [4–7].

Resveratrol (RSV; 3,5,4′-trihydroxystilbene) is a natural polyphenolic phytoalexin produced in plants. RSV can alter the activities of the histone deacetylases (HDACs) I and II, DNA methyltransferases (DNMTs) and is an activator of Sir2, a sirtuin belonging to HDACIII [6]. Sir2 modulates the acetylation status of H4K16 [8, 9]. RSV acts as an antioxidant at low doses, but it is a pro-oxidant and generates DNA damage at high doses [10]. Treatment of human cell lines with RSV induces Rad9 expression, a key player in the DNA damage response (DDR), as well as H2A.X phosphorylation at the SQE motif (γH2A.X), a double strand break (DSB) marker [11].

RSV administration during acute *T. gondii* infection in mice would confer protection of the host [12], regulate tissue inflammation and reduce parasite replication [13]. Chen et al. [14] observed that the incubation of extracellular tachyzoites with RSV for 24 h affected their viability. They also observed that RSV reduced tachyzoite intracellular growth and promoted autophagy in infected macrophages. Adeyemi et al. [15] identified RSV as a putative drug candidate against toxoplasmosis. However, no studies have investigated the mechanism(s) by which RSV affects *T. gondii* tachyzoites.

In this work, we evaluated the effects of RSV on *T. gondii* replication and histone posttranslational modification (PTM) and its effects on H3 and H4K16 acetylation levels. In addition, we investigated whether γH2A.X levels differ between control and treated intracellular tachyzoites. Collectively, our results show that RSV inhibited *T. gondii* replication and induced H4K16 and H3 deacetylation. Furthermore, γH2A.X levels were highly increased in tachyzoites treated with RSV relative to control tachyzoites, suggesting an association between RSV treatment and DSB damage.

## Main text

### Materials and methods

#### Parasite sources, culture and reagents

Tachyzoites of RH wild-type and RH RFP (Red Fluorescent Protein) strains were cultured under standard conditions in vitro in monolayers of immortal human foreskin fibroblasts (hTERT, ATCC^®^ CRL-4001, USA). The RH RFP was kindly provided by Dr. Silvia N. Moreno (University of Georgia, Athens, GA, USA). Cell monolayers were infected with tachyzoites and incubated with high-glucose Dulbecco’s modified Eagle medium (DMEM, Invitrogen, Argentina) supplemented with 10% (10X) or 1% (1X) fetal bovine serum (FBS, Internegocios S.A., Argentina) and penicillin (10,000 units/ml)-streptomycin (10 mg/ml) solution (Gibco, Argentina) at 37 °C in a 5% CO_2_ atmosphere. Resveratrol (Abcam, 120,726, USA) solution was prepared in DMSO (Sigma, Argentina) vehicle and stocked at 10 mg/ml.

Commercial rabbit antibodies anti-H3 (Abcam ab10799, USA), anti-H3ac (Millipore, 06-599B, USA), anti-H4 (Abcam ab31830, USA) and anti-H4K16ac (Abcam, ab109463, USA) were used as primary antibodies, and Alexa Fluor goat anti-mouse 488 (Invitrogen, A11001, Argentina) and anti-rabbit 595 (Invitrogen, A11037, Argentina) were used as secondary antibodies. Alkaline phosphatase–conjugated antibodies were used in Western Blot assays (Santa Cruz Biotechnology, Argentina).

A serum sample of rabbit anti-*T. gondii* γH2A.X was obtained from Eurogentec (Belgium) based on the peptide NH_2_-C + GKHGV-S_(PO3H2)_-QEF–COOH, designed from the *T. gondii* H2A.X amino acid sequence. Rabbit anti-rH2A.X was obtained from the Animal Facility at Facultad de Ciencias Exactas y Naturales (University of Buenos Aires, Argentina) on the basis of the recombinant rH2A.X [16]. Murine anti-SAG1 was kindly provided by Dr. Marina Clemente [17].

#### Toxicity assay

To evaluate the cytotoxic effect of the drugs on host cells, fibroblasts (hTERT) were seeded at 40% confluence (1.6 × 10^4^ cells/well) in 96-well plates and incubated for 24 h. After this period, confluence was assessed by microscopy, and the medium was replaced with fresh medium containing RSV at one of several concentrations. For the experiments in which the cells were incubated with RSV for 24 h, the concentrations ranged between 0 and 200 µM. Cell viability was determined based on the reduction of 3-(4,5 dimethyl-2-thiazoyl)-2,5-diphenyltetrazole bromide (MTT, Sigma). The absorbance at 540 nm was measured from the bottom of the plate using a BioTek Synergy plate reader (BioTek, Argentina). The graphs display the viability of the cells relative to the viability of control cells treated with 0.5% DMSO in culture media (100% viability).

#### Replication assay

Replication was performed and assessed using RFP-expressing RH tachyzoites as previously described [18]. For intracellular exposure, hTERT cells were infected with 10,000 RH RFP parasites. The other half of the plate was used for control treatment (host cells + RSV of different concentration). After 3 h of infection, the medium was replaced with 1X fresh medium containing one of several concentrations of RSV or vehicle. The parasites were then allowed to replicate for 24 h on the host cells. Relative fluorescent unit (RFU) values of RFP were collected using a Synergy H1 Hybrid Multi-Mode Microplate Reader (Biotek, Argentina). Measurements at each concentration of RSV were collected in triplicate. The basal fluorescence was estimated as 234 ± 2.3 RFU. We also calculated the IC_50_ value with GraphPad Prism 6 (San Diego, USA) following previously described methods [19]. Data were normalized with 0 as the smallest value and transformed to a semilogarithmic scale (x = log(x)). Then, the data were analyzed as a nonlinear regression parameter: dose–response inhibition-log(inhibitor) vs normalized response-variable slope.

#### Indirect immunofluorescence assay (IFA)

Intracellular tachyzoites were incubated for 24 h with 100 μM RSV, washed with 1X PBS and fixed with methanol for 10 min at −20 °C. Then, the cells were permeabilized with 0.2% v/v Triton X-100 in PBS for 10 min and blocked with 3% w/v BSA in PBS for 30 min at room temperature (r.t.). Then, primary antibodies diluted in 0.5% w/v BSA in PBS were added, and the cells were incubated for 60 min at r.t. The cells were then washed three times with PBS and then incubated with the corresponding Alexa Fluor-conjugated secondary antibodies for 60 min at r.t. Samples were mounted on coverslips with mounting medium (Mowiol 4-88, Sigma, Argentina) containing 10 µg/µl DAPI (4,6-diamidino-2-phenyl-indole) to stain the nuclei. Primary antibodies were diluted 1:200, whereas secondary antibodies were diluted 1:2000. Samples were analyzed by epifluorescence microscope (Zeiss Axioscope, Germany) equipped with a 63X objective and a Zeiss Axiocam 506 mono microscope camera. Images and fluorescence intensity analysis were obtained with the Fiji package of ImageJ (NIH, USA).

#### Western blot

Immunoblotting was performed as previously described with minor modifications [16]. Intracellular parasites were incubated for 24 h with RSV. To obtain large quantities of tachyzoites, doses of 50–70 μM were tested. Proteins were extracted from purified parasites (10^7^ parasites/lane). Nonspecific binding sites were blocked with 5% nonfat dry milk in TBS containing 0.1% v/v Tween-20 (TBS-T) for 6 h, and the membranes were then incubated overnight with primary antibodies: mouse anti-H4, rabbit anti-H4K16ac, mouse anti-H3, rabbit anti-H3ac, rabbit anti-γH2A.X, rabbit anti-H2A.X and mouse anti-SAG1. Band intensities were quantified from scanned images using ImageJ software, and the value obtained from each band was normalized to the loading control, SAG1. Then, each histone PTM was normalized to its respective histone (H2A.X or H4).

#### Statistics

Data were expressed as mean ± SD from three to four experiments. The variations in the data were analyzed using unpaired Student´s *t* test (*p ≤ 0.05 and ****p ≤ 0.0001) with GraphPad Prism 6.1 software (San Diego, USA).

### Results and discussion

**Fig. 1:**
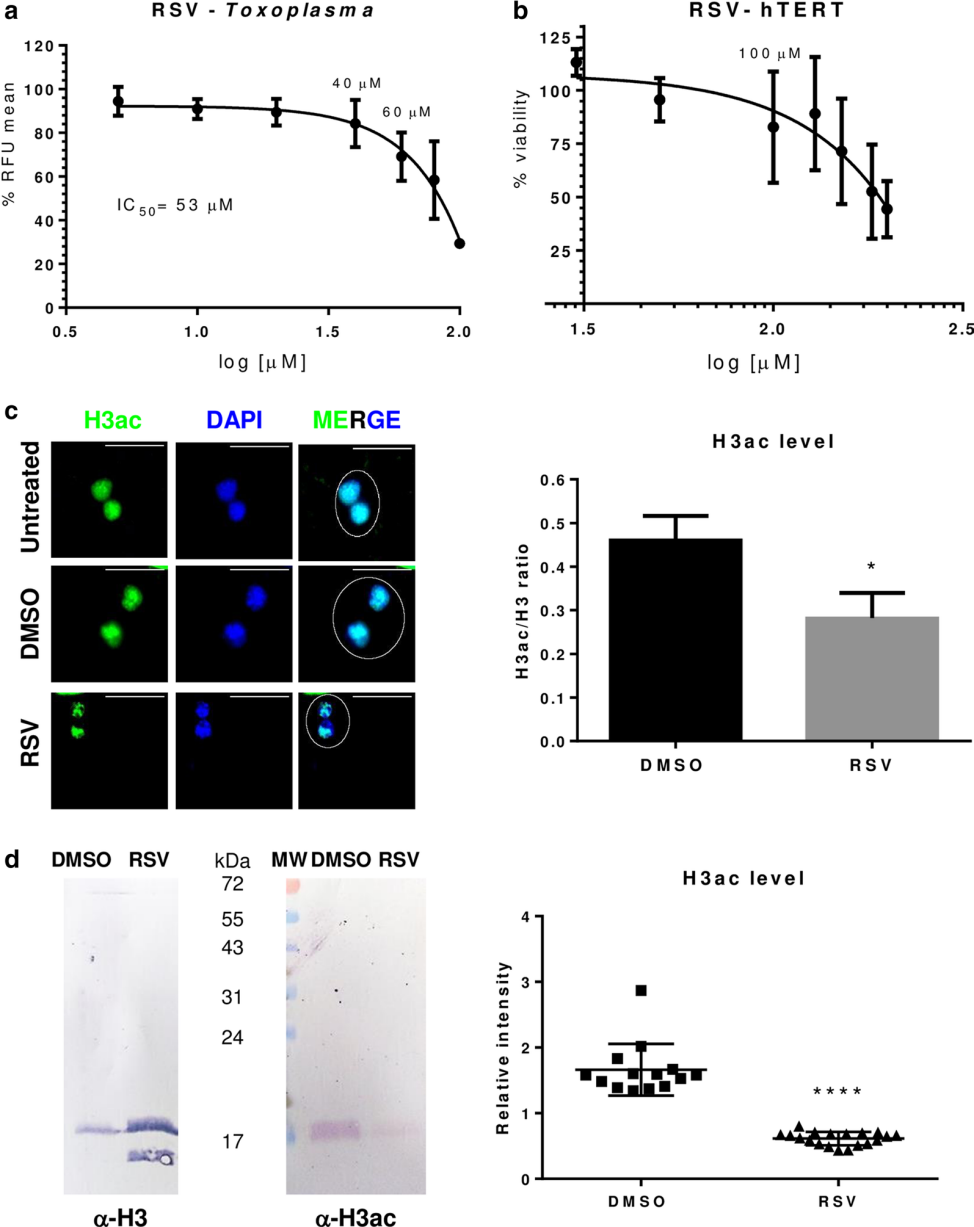
Effects of resveratrol (RSV) on *T. gondii* replication and H3 acetylation **a** Effect of RSV on intracellular RH tachyzoite growth. A dose-dependent growth (0-100 μM) curve relative to RFP fluorescence after 24 h of treatment is shown. Three independent experiments were performed in triplicate each time, and the data are presented as mean ± standard deviation (SD). **b** hTERT cell monolayers were incubated for 24 h in the absence or presence of RSV. Cytotoxicity was estimated by 3-(4,5-dimethylthiazol-2-yl)-2,5-diphenyl-tetrazolium bromide reduction (with absorbance measured at 540 nm), which is presented relative to the viability of the untreated controls (defined as 100% viability). Determinations were performed in triplicate. The results are representative of three independent experiments. Untreated controls were incubated with 0.5% v/v DMSO. **c** Indirect immunofluorescence with anti-H3ac antibody (1:200). PV with two parasites were chosen to compare treatment and vehicle (white circle). The upper right white lines correspond to the scale bar: 10 µm. Nuclei were stained with DAPI. Intracellular tachyzoites were treated with RSV (100 μM) or DMSO 0.5% for 24 h. DAPI and fluorescence intensities were quantified from 10 nuclei (Additional file 1) and plotted as relative intensities. The panel and graph are representative of three independent experiments with similar results. **d** Western blot of *T. gondii* lysates with anti-H3 (1:250) or anti-H3ac (1:500) antibodies. Lysates were obtained from purified intracellular tachyzoites treated with RSV (50-70 μM) or DMSO 0.5% for 24 h. H3ac band intensities were quantified relative to histone H3 band intensity and plotted. Statistical analysis was performed with one-way ANOVA and Tukey’s multiple comparison test (*p ≤ 0.05 and ****p ≤ 0.0001)

To test our study model, uninfected or infected hTERT host cells were incubated for 24 h with different doses of RSV (Fig. 1a, b). Nearly 80% of the uninfected hTERT cells remained viable at an RSV concentration of 100 μM (Fig. 1b). To determine the impact of RSV on the *T. gondii* lytic cycle, transgenic RH RFP tachyzoites were grown intracellularly for 24 h in the presence of RSV. RSV showed a dose-dependent effect on intracellular *T. gondii* replication (Fig. 1a), yielding an IC_50_ value of 53 ± 4 μM. These results agree with those of Chen et al. [14], who found that 50 μM RSV blocked *T. gondii* intracellular growth. Adeyemi et al. [15] identified RSV as one of 62 compounds with anti-*T. gondii* effects, which yielded an IC_50_ value of 1.03 μg/ml (4.4 μM) after 72 h of incubation in comparison with pyrimethamine. The authors also observed that HFF cells were 100% viable at an RSV concentration of 2 μg/ml (8.5 μM). The differences in IC_50_ values among studies may be due to differences in the duration of drug exposure.

**Fig. 2:**
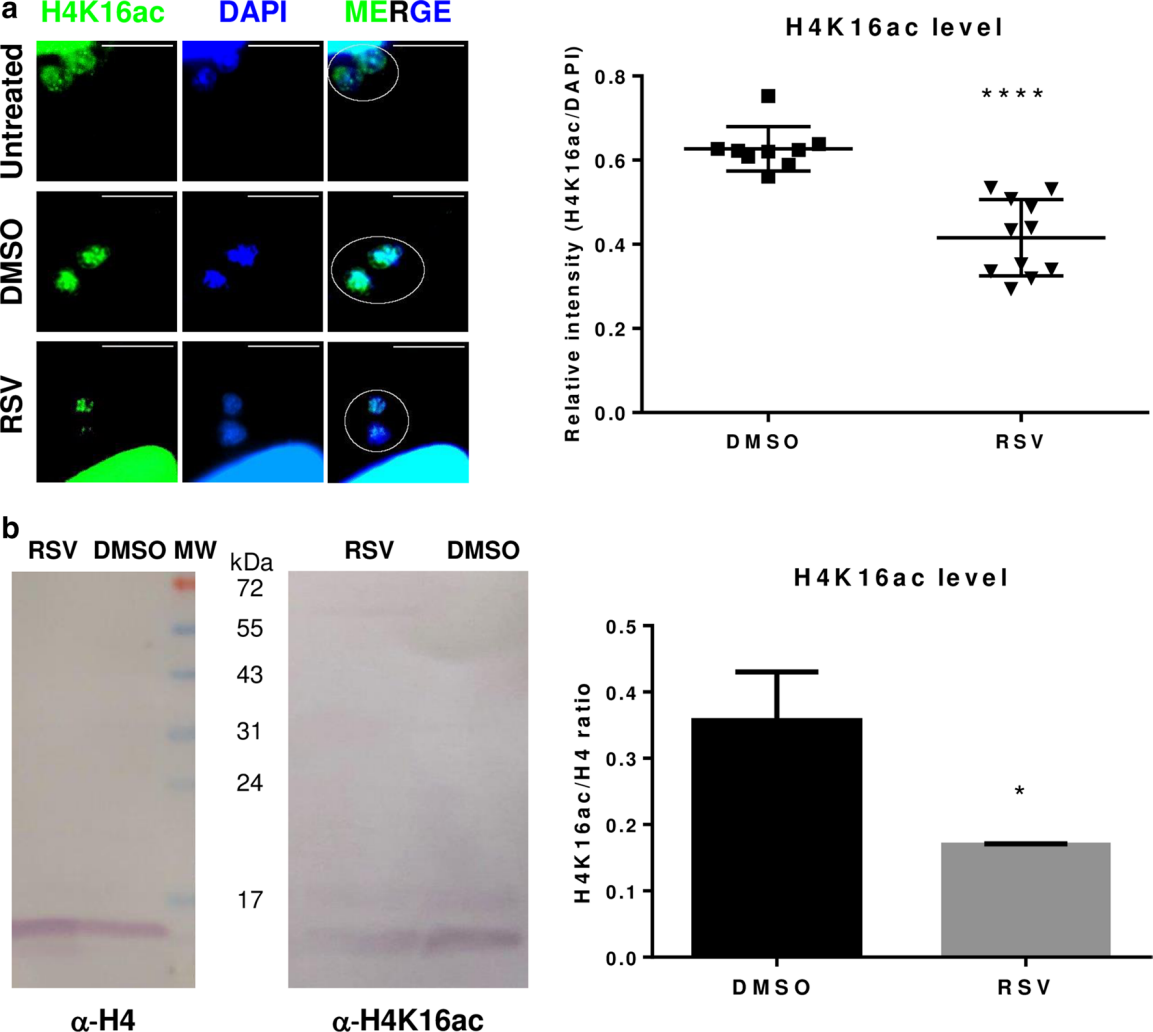
Effect of resveratrol (RSV) on *T. gondii* H4K16 acetylation **a** Indirect immunofluorescence with anti-H4K16ac antibody (1:200). PV with two parasites were chosen to compare treatment and vehicle (white circle). The upper right white lines correspond to the scale bar: 10 µm. Nuclei were stained with DAPI. Intracellular tachyzoites were treated with RSV (100 μM) or DMSO 0.5% for 24 h. DAPI and fluorescence intensities were quantified from 10 nuclei (Additional file 1) and plotted as relative intensities. The panel and graph are representative of three independent experiments with similar results. **b** Western blot of *T. gondii* lysates with anti-H4 (1:250) or anti-H4K16ac (1:500) antibodies. Lysates were obtained from purified intracellular tachyzoites treated with RSV (50 μM) or DMSO 0.5% for 24 h. H4K16ac band intensities were quantified relative to histone H4 band intensity and plotted. Statistical analysis was performed with one-way ANOVA and Tukey’s multiple comparison test (*p ≤ 0.05 and ****p ≤ 0.0001)

Since HDAC enzymes are one of the multiple targets of RSV, we analyzed the acetylation levels of H3 and H4K16. Tachyzoites treated with RSV presented significantly weaker H3ac and H4K16ac labeling (p ≤ 0.05 and p ≤ 0.0001, respectively) than tachyzoites treated with DMSO (Figs. 1c and 2a and Additional file 1). Untreated and DMSO controls did not differ in their fluorescence intensities (Figs. 1c and 2a). We decided to corroborate these data by Western blot. Compared to treatment with the DMSO control, RSV treatment reduced the H3ac and H4K16ac mark intensities at minimum doses of 70 μM (p ≤ 0.0001) and 50 μM (p ≤ 0.05), respectively (Figs. 1d and 2b). These results are in agreement with a previous study in which RSV treatment was found to decrease H3 and H4 acetylation levels in *Trypanosoma cruzi* [19]. Furthermore, it was recently observed that RSV treatment reduced H4K16ac marker intensity [20]. In addition, in yeast, Sir2, a target of RSV, was found to negatively control the activation of DNA replication origins within heterochromatin and euchromatin by deacetylating H4K16 [21]. Our results suggest that RSV potentially affects *T. gondii* transcription and/or replication through H3 and H4K16 deacetylation. However, further studies are required to confirm this possibility.

**Fig. 3:**
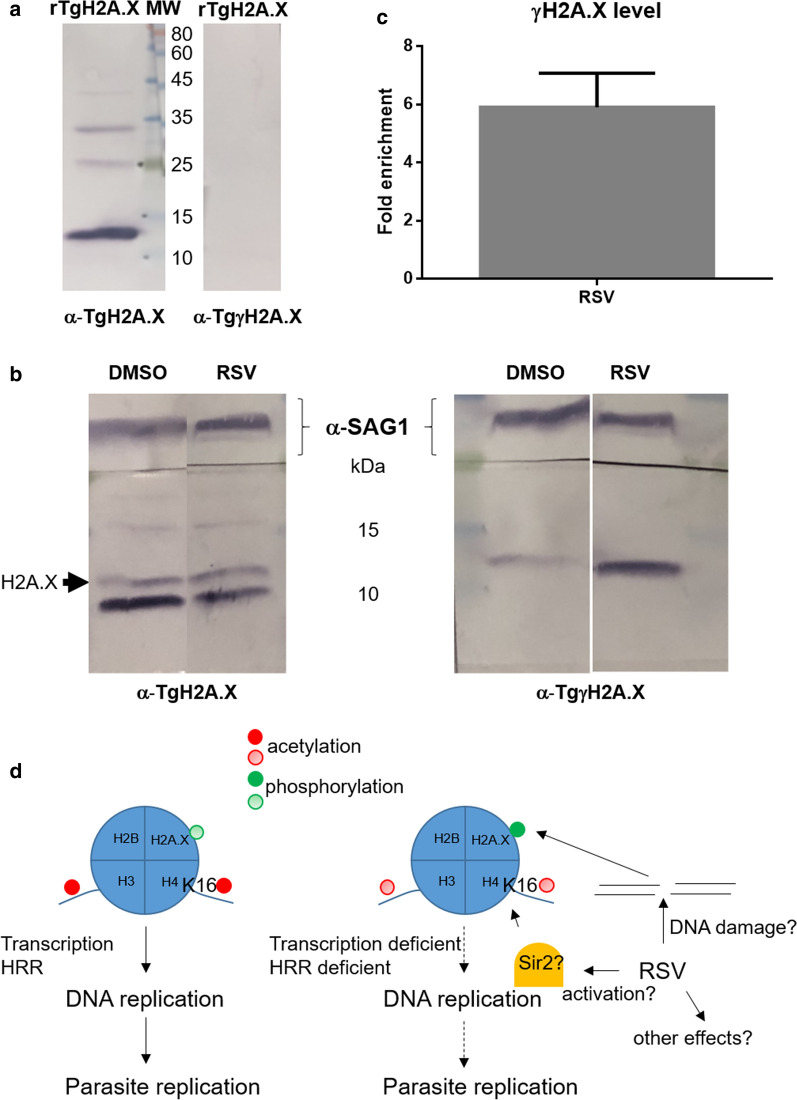
Effect of intracellular exposure to resveratrol (RSV) on *T. gondii* H2A.X phosphorylation (γH2AX). **a** Western blot of *T. gondii* recombinant H2A.X (rH2A.X; 200 ng/lane) expressed in *Escherichia coli* and purified by nickel resin. Rabbit anti-rTgH2A.X (α-TgH2A.X, 1:5000) and anti-*T. gondii* phosphorylated peptide (α-TgγH2A.X, 1:100). The phosphorylated peptide sequence was NH_2_- C + GKHGV-S_(PO3H2)_-QEF -COOH. **b** Western blot of *T. gondii* lysates with anti-SAG1 (*T. gondii* surface antigen 1, 1:500), anti-TgH2A.X or anti-TgγH2A.X. Lysates were obtained from purified intracellular tachyzoites previously treated with RSV (50 μM) or DMSO 0.5% for 24 h. H2A.X (arrow) and γH2A.X band intensities were quantified and normalized to SAG1 band intensities. The relative intensity of the bands (γH2A.X/H2A.X) was then calculated for each treatment. The signal normalized to SAG1 was calculated from relative values in comparison to the DMSO control. The results are the means of three replicates ± SD. This panel corresponds to Experiment 3 in Additional file 2. **c** Quantitation of the fold increase in γH2A.X level after RSV treatment. **d** Putative model of RSV action that leads to a decrease in *T. gondii* replication. Dotted lines indicate reduced activities or processes. For acetylations and phosphorylations, color intensity represents the PTM mark level

To determine whether the effect of RSV is associated with DSB damage, the level of γH2A.X (a marker of DSB level) was determined by Western blot. A specific rabbit anti-*T. gondii* phosphorylated peptide in the SQE C-terminal motif of *T. gondii* H2A.X was prepared. To confirm its specificity, a Western blot assay against recombinant nonphosphorylated *T. gondii* H2A.X was performed. Rabbit anti-TgγH2A.X antibody did not recognize *T. gondii* H2A.X (Fig. 3a) but reacted with an expected band of 14.5 kDa in *T. gondii* lysate (Fig. 3b). After treatment with RSV, the γH2A.X signal was increased compared to that attained under treatment with DMSO control (Fig. 3b and Additional file 2), by approximately 5.90 times (Fig. 3c).

In the present study, we found that the level of γH2A.X, a marker of DNA damage, is increased in the presence of RSV. In another model, 50 µM RSV induced DNA damage and S-phase arrest and enhanced γH2A.X levels in a panel of head and neck squamous cell carcinoma lines [22]. Double strand breaks (DSBs) are repaired by homologous recombination (HRR), which is triggered in late S phase by DSBs that occur during DNA replication (e.g., due to fork collapse) or nonhomologous end joining (NHEJ) throughout the cell cycle [6]. Histone H4 acetylated on its lysine at position 16 (H4K16ac) is a modification that facilitates activates the HRR pathway [6, 23]. Considering all of the results together, we propose a possible model of RSV action at the concentrations studied here that can partially explain RSV’s effect on *T. gondii* replication (Fig. 3d). Briefly, RSV might induce a deficiency in H3 and H4K16 acetylation, thereby leading to altered transcription levels, the initiation of DNA replication and, potentially, activation of the HRR pathway. These processes might then affect the accuracy of DNA replication and the progression of the cell cycle, maintaining γH2A.X at high levels. In turn, RSV might induce DNA damage, possibly by oxidation, generating fork collapse and DSBs [24, 25] and thereby increasing the difficulty of repair via HRR in the absence of H4K16ac.

### Limitations

Since RSV is a multitarget drug, it is difficult to establish which targets in *T. gondii* are affected by RSV. One of the targets might be a histone acetyltransferase. However, RSV shows sirtuin-activating activity [26]. Thus, it is possible that sirtuins are among the RSV targets leading to histone deacetylation. *T. gondii* sirtuins Sir2a and Sir2b have not yet been characterized. Therefore, their specific functions (e.g., their roles in the acetylation of H4K16), genomic localization when present in the nucleus, etc., have not yet been reported. This lack of knowledge impairs our ability to infer whether the observed effects of RSV are associated with these sirtuins. Our results and those of others show that RSV exhibits anti-*T. gondii* activity in vitro. Studies in mice should be conducted to further evaluate RSV’s potential as a therapeutic candidate against toxoplasmosis. Very importantly, at low doses, RSV has beneficial effects on a wide variety of human diseases; however, at high doses and under long-term application, it can have adverse effects [10]. This aspect should be taken into account when considering an RSV-based anti-*T. gondii* therapy. Here, we observed that in vitro, the IC_50_ of RSV was lower than the threshold of hTERT toxicity. Perhaps the dose and treatment duration could be managed by combining RSV with other drugs (e.g., HRR inhibitors) to achieve a synergistic effect.

## Supplementary information

**Additional file 1:** Quantification of H4K16ac mark with or without RSV treatment. The panels show ten nuclei of intracellular tachyzoites under RSV or DMSO control treatment labeled by indirect immunofluorescence with α-H4K16ac (1:200). Nuclei were also stained with DAPI. Antibody fluorescence and DAPI signals were quantified and plotted in different graphs (see Fig. 3).

**Additional file 2:** Detection of γH2A.X under different treatments. Western blot of *T. gondii* lysates performed as described in the Materials and Methods section and revealed with anti-SAG1 (*T. gondii* surface antigen 1, 1:500), anti-TgH2A.X (1:5000) or anti-TgγH2A.X (1:100). Lysates were obtained from purified intracellular tachyzoites previously treated with RSV 50 μM (R), Sirtinol 50 μM (S) or DMSO 0.5% v/v (C) for 24 h. Three independent experiments with similar results are shown.

## Data Availability

All the raw data generated are available upon reasonable request to the corresponding author.
