# Supplementary figures and images for: Resveratrol induces H3 and H4K16 deacetylation and H2A.X phosphorylation in Toxoplasma gondii
Source: BMC Res Notes. 2021 Jan 7;14:19. doi: 10.1186/s13104-020-05416-4 (PMC7792170; doi:10.1186/s13104-020-05416-4)

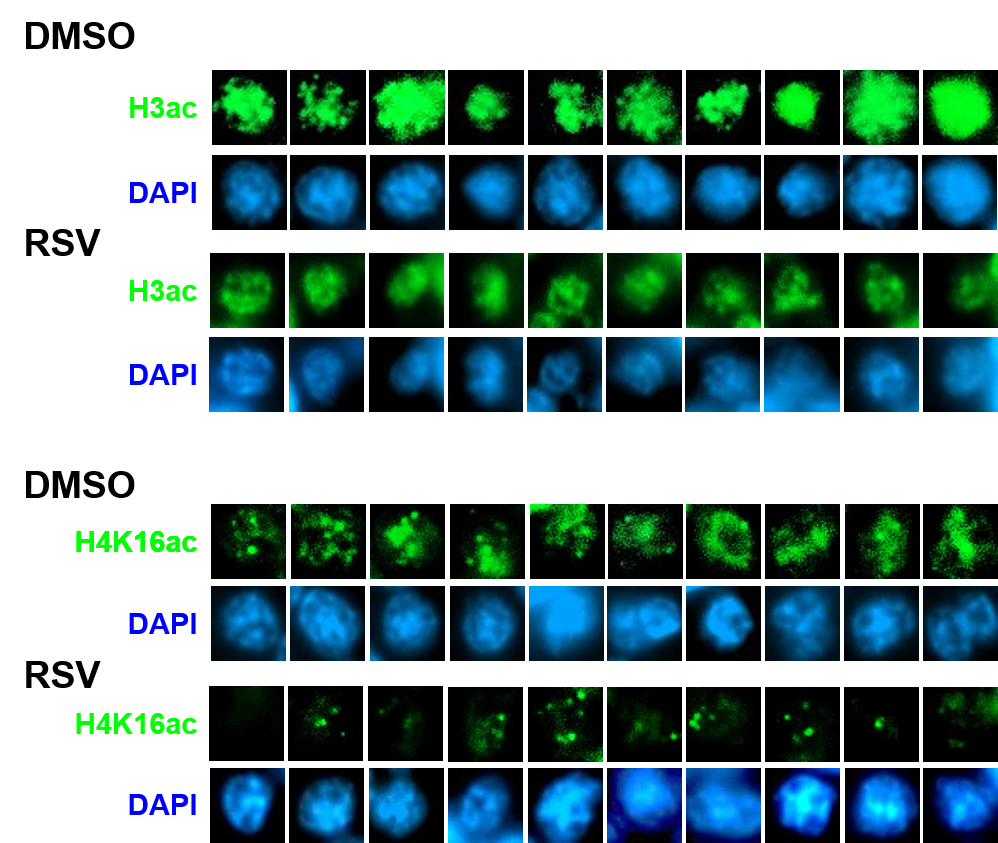

Supplement: Supplementary file 1 — Additional file 1: Quantification of H4K16ac mark with or without RSV treatment. The panels show ten nuclei of intracellular tachyzoites under RSV or DMSO control treatment labeled by indirect immunofluorescence with α-H4K16ac (1:200). Nuclei were also stained with DAPI. Antibody fluorescence and DAPI signals were quantified and plotted in different graphs (see Fig. 3). [file 13104_2020_5416_MOESM1_ESM.tif]

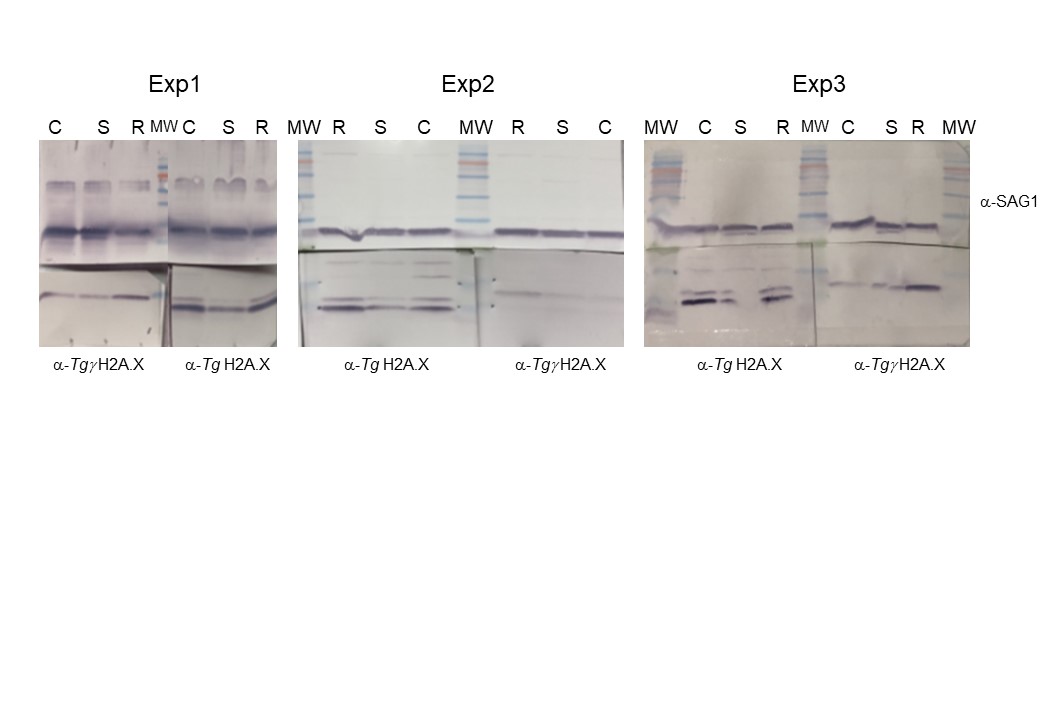

Supplement: Supplementary file 2 — Additional file 2: Detection of γH2A.X under different treatments. Western blot of T. gondii lysates performed as described in the Materials and Methods section and revealed with anti-SAG1 (T. gondii surface antigen 1, 1:500), anti-TgH2A.X (1:5000) or anti-TgγH2A.X (1:100). Lysates were obtained from purified intracellular tachyzoites previously treated with RSV 50 μM (R), Sirtinol 50 μM (S) or DMSO 0.5% v/v (C) for 24 h. Three independent experiments with similar results are shown. [file 13104_2020_5416_MOESM2_ESM.jpg]
